# Congressional Appropriations for Medical and Health Purposes

**Published:** 1882-07

**Authors:** 


### Editorial.

---

### CONGRESSIONAL APPROPRIATIONS FOR MEDICAL AND HEALTH PURPOSES.

The action of the present Congress in reducing the appropriation of money for carrying on the work of the National Board of Health, to so small a sum as to suspend much of the most important work of that board, is a striking illustration of Congressional ideas of political economy. We are no advocates of national centralization, or of calling on the general government to do what can be better and more economically done by the state or local municipalities. But a former Congress deemed it wise to create a National Board of Health, and to assign to it duties of importance to the whole people. During the brief period since its creation, the Board has certainly done much useful work, and has barely had time to mature plans for accomplishing much more, when it finds itself seriously crippled by an unreasonable reduction of the annual appropriation. The same spirit prompted the committee of one house to report in favor of reducing the ordinary appropriation for the library and museum of the Surgeon General's office from $10,000 to $5000, which, if finally adopted, would arrest the publication of the *Index*, and permanently injure both those important interests. If the national revenues were deficient, or if the same apparent economical spirit was manifested in regard to all the items contained in the appropriation bills passed by Congress, we might respect the motives of its members, however much we might think they erred in judgment. But when we find items in the same appropriation bills amounting to millions of dollars, freely voted, for objects of no national importance, we can neither respect their consistency nor their ideas of economy. And when we look further and find that a majority of the members of the same Congress have deliberately voted more money, taken from the hard earnings of the American people, to pay for the *liquors and cigars* of one Congressional debauch, under the pretense of a battle-field celebration, than they are willing to vote for the support of two as important and permanent educational institutions as the Army Medical Museum and Library, we confess to a feeling even beyond that which is expressed by the word disgust. It is quite possible that the time may come when members of Congress will learn that the greater part of the sixty thousand or seventy thousand practitioners of medicine in the United States are voters.

